# Study of Imaging Analysis for Distinguishing Alveolar Collapse From Invasive Lesions in Early‐Stage Lung Adenocarcinoma

**DOI:** 10.1111/1759-7714.70246

**Published:** 2026-01-15

**Authors:** Shinji Kaneda, Atsushi Ito, Daisuke Ito, Teruhisa Kawaguchi, Motoshi Takao, Koji Kawaguchi

**Affiliations:** ^1^ Department of Thoracic Surgery Mie University Graduate School of Medicine Tsu Japan; ^2^ Department of Respiratory Center, Matsusaka Municipal Hospital Matsusaka Japan

**Keywords:** alveolar collapse, early‐stage lung adenocarcinoma, three‐dimensional volumetric analysis

## Abstract

**Background:**

Non‐invasive lung adenocarcinoma may occasionally contain solid benign components, presenting challenges for making an accurate diagnosis using CT (computed tomography). Therefore, we investigated whether a three‐dimensional (3D) volumetric analysis was more effective than CT in distinguishing the presence of invasive lesions in early‐stage lung adenocarcinoma.

**Materials and Methods:**

We retrospectively classified 161 patients preoperatively diagnosed with clinical stage IA1 into two groups: the non‐invasive lesion group (50 patients) and the invasive lesion group (111 patients). We conducted a comparative analysis concerning the predictive performance of CT and a 3D volumetric analysis to identify invasive lesions. In addition, a histological review of the pre‐invasive lesion group was performed to assess the pathological characteristics of the solid component, as observed on preoperative CT.

**Results:**

A univariate analysis demonstrated that the consolidation diameter, consolidation/tumor (C/T) ratio, consolidation volume, and three‐dimensional (3D)‐C/T ratio were significant predictors of the presence of invasive lesions. A multivariate analysis identified the consolidation volume as an independent predictor (*p* = 0.045). Analyses of the receiver operating characteristics showed that the areas under the curve for the consolidation diameter, consolidation volume, C/T ratio, and 3D‐C/T ratio were 0.702, 0.634, 0.747, and 0.742, respectively. In addition, a histological review revealed alveolar collapse in 89.2% (25 of the 28) of the preinvasive lesion group.

**Conclusions:**

Consolidation diameter, consolidation volume, and 3D‐C/T ratio are more reliable predictors for distinguishing alveolar collapse from invasive lesions than the C/T ratio in early‐stage lung adenocarcinoma.

## Graphical abstract

Consolidation diameter, consolidation volume, and 3D‐C/T ratio are more reliable predictors for distinguishing alveolar collapse from invasive lesions than the C/T ratio in early‐stage lung adenocarcinoma. Importantly, accurately distinguishing alveolar collapse from invasive lesions may contribute to more appropriate therapeutic decision‐making and ultimately improve patient outcomes.

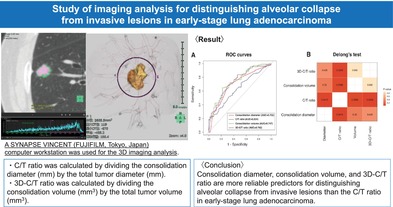

## 1 Introduction

Recent advances in imaging modalities and the widespread adoption of chest computed tomography (CT) screening have contributed to the growing rate of early detection of pure ground‐glass opacities (GGOs) and part‐solid ground‐glass nodules (GGNs). Pure GGOs represent localized nodular areas with increased lung attenuation, whereas part‐solid GGNs consist of a combination of ground‐glass and solid components [1]. These findings cover a broad spectrum of pathologies, ranging from non‐invasive lesions, such as atypical adenomatous hyperplasia (AAH) and adenocarcinoma in situ (AIS), which typically appear as pure GGOs on CT, to minimally invasive adenocarcinoma (MIA) and invasive adenocarcinoma (IA), which are often characterized by part‐solid GGNs or pure‐solid nodules. However, non‐invasive lesions may occasionally contain solid components [2].

The solid components within these nodules can correspond to invasive lesions or benign conditions, such as alveolar collapse, inflammation, or mucin accumulation [3, 4]. The association between alveolar collapse and lung adenocarcinoma was first described in 1980 by Shimosato et al., who identified a distinct growth pattern [5]. Subsequently, in 1995, Noguchi et al. categorized tumors in peripheral small lung adenocarcinoma into three types based on their histological characteristics: type A, characterized by the retention of alveolar structures; type B, marked by fibrotic foci due to alveolar collapse; and type C, displaying active fibroblastic proliferation [6]. Among them, type A and B tumors do not show lymph node metastases, have rare vascular and pleural invasion, and exhibit an excellent prognosis with a 5‐year survival rate of 100% [6]. In contrast, type C tumors are considered advanced carcinomas with invasive fibroblast proliferation, showing a 5‐year survival rate of 74.8% [6]. Therefore, distinguishing the benign alveolar collapse from invasive lesions in early‐stage adenocarcinoma is crucial because of the significant differences in the prognosis. However, both are visually recognized as solid components on CT, posing a challenge in distinguishing them preoperatively.

In recent years, considerable attention has been paid to three‐dimensional (3D) volumetric analyses for assessing the invasiveness of lung adenocarcinoma [7, 8, 9, 10]. The measurement of solid components on CT is occasionally problematic because of the irregular shape of the tumor. Such irregularly shaped tumors may require an evaluation from multiple axes, which may mislead physicians regarding the clinical diagnosis of the tumor diameter. In contrast, a 3D volumetric analysis allows for the direct assessment of the tumor and an evaluation over all axes, ensuring an accurate assessment, regardless of the shape [8]. Several recent studies have suggested that a 3D volumetric analysis is superior to conventional CT in its ability to predict biological malignancy and the prognosis in patients with early‐stage lung cancer [7, 8, 9, 10]. However, whether a 3D volumetric analysis can effectively differentiate between alveolar collapse and invasive lesions in early‐stage lung adenocarcinoma remains unclear.

Therefore, we investigated the potential advantages of 3D volumetric analysis for distinguishing the presence of invasive lesions in early‐stage lung adenocarcinoma compared with the conventional CT image. We classified 161 patients diagnosed with clinical stage IA1 adenocarcinoma preoperatively into 2 groups: the preinvasive lesion (AAH‐AIS) group and the invasive lesion (MIA‐IA) group. Subsequently, we conducted a comparative analysis to assess the predictive performance of CT and a 3D volumetric analysis for detecting the presence of invasive lesions. Furthermore, we performed a histologic review of the 28 patients in the non‐invasive lesion group to assess the pathological characteristics of the solid components, as observed on preoperative CT.

## 2 Materials and Methods

### 2.1 Study Population

In this retrospective study, we performed radiological and pathologic revisions in a cohort of 703 patients who underwent lung resection for primary lung cancer in the Department of Thoracic and Cardiovascular Surgery, Mie University Graduate School of Medicine, Japan, between January 1, 2019, and December 31, 2024. The TNM stage was determined based on the eighth edition of the TNM classification for malignant tumors. We excluded 186 patients who were diagnosed with histological types other than adenocarcinomas. Of the remaining 517 patients, we focused on the 161 with a preoperative diagnosis of clinical stage IA1. Subsequently, we classified these patients into the 50 non‐invasive lesion group (AAH‐AIS) and 111 the invasive lesion group (MIA‐IA) based on pathological findings. In patients with multiple synchronous lung cancers (*n* = 4), the tumor with the highest‐grade was included in the analysis.

**FIGURE 1:**
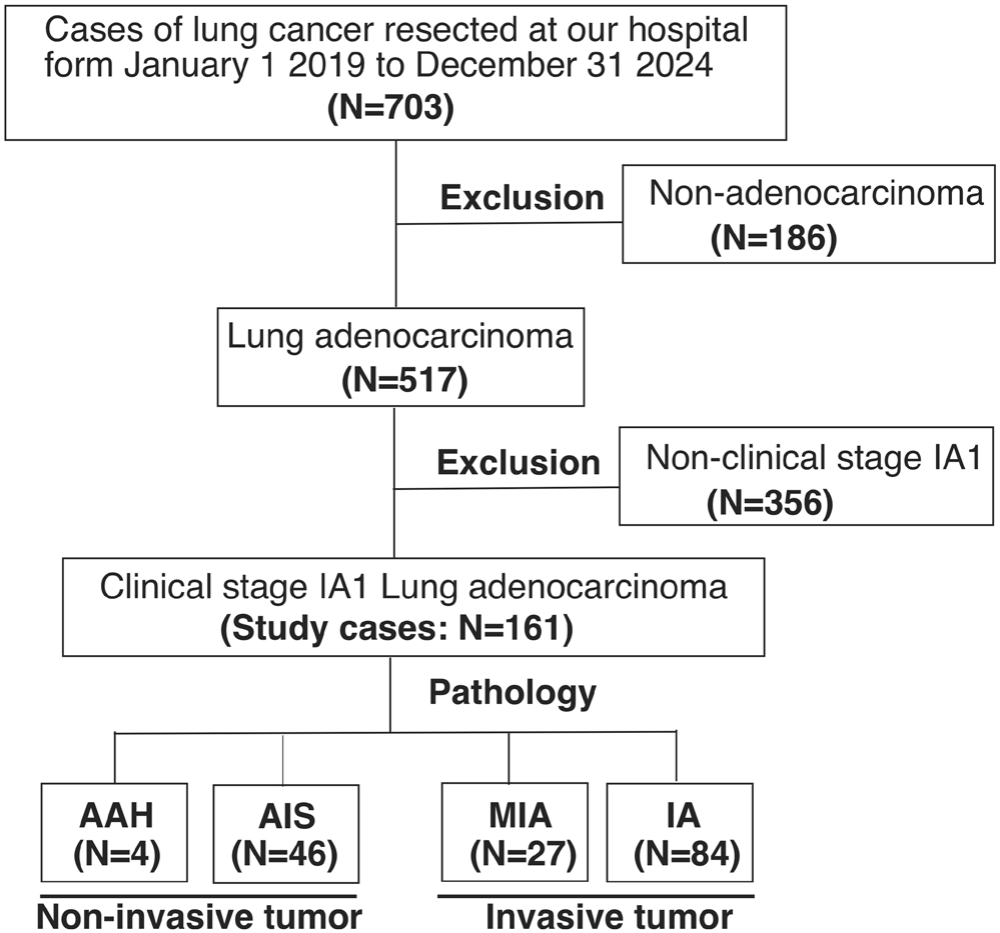
Study the patient selection process. AAH, atypical adenomatous hyperplasia; AIS, adenocarcinoma in situ; MIA, minimally invasive adenocarcinoma; IA, invasive adenocarcinoma; *N*, number.

The patient selection process is illustrated in Figure 1. A comprehensive review of the medical records was conducted for all patients to gather information on the age, sex, smoking history, serum carcinoembryonic antigen (CEA), and histopathological data. Radiological parameters, including the maximum tumor diameter, consolidation diameter, consolidation/tumor (C/T) ratio, maximum tumor volume, consolidation volume, 3D‐C/T ratio, and maximum standardized uptake value (SUV_max_) with 2‐[^18^F]‐fluoro‐2‐deoxy‐d‐glucose (FDG)‐positron emission tomography (PET), were also examined. The numbers of missing data were 40 for smoking history, 60 for PET, 36 for CEA, and two for surgical procedures. The clinical characteristics and radiological findings were retrospectively compared between the non‐invasive and invasive lesion groups. This study was approved by the Research Ethics Board of Mie University School of Medicine (H2021‐133).

### 2.2 Radiologic Examinations

CT was conducted using 64‐, 192‐, 256‐, and 320‐row multisection helical CT systems (GE Healthcare, Siemens, Siemens, Canon). Standard contrast‐enhanced imaging protocols were used to assess the lung apex to the upper abdomen with these parameters: 120‐kV peak, tube current‐time product 120–200 mA, beam pitch 0.5 (Siemens, Canon) or 0.625 (GE Healthcare), 512 × 512‐pixel resolution, 0.5‐s scan time. Axial images were reconstructed at a cross‐sectional thickness of 1 mm. Images were displayed at 500–700 HU, window width 1000–2000 HU (lung window setting) and 30–60 HU, window width 350–600 HU (mediastinal window setting).

### 2.3 Radiologic Evaluations

The maximum tumor diameter was defined as the longest diameter, including GGO lesions, in the lung window settings. GGO refers to localized areas of increased attenuation that do not obscure lung vessels when viewed from a lung window setting. All preoperative CT data were transferred to a computer workstation (SYNAPSE VINCENT version 6.0; FUJIFILM, Tokyo, Japan) equipped with the ability to display a CT density profile across the tumor. The 3D shapes of the tumors were semi‐automatically visualized by tracking the edges of the pure‐solid or part‐solid GGN across all axial images. Furthermore, any structures that overlapped with the GGN, such as pulmonary vessels, were excluded from the calculation target by manually drawing the region of interest (ROI) on the axial image. An ROI was drawn freehand around the tumor using an electronic cursor and mouse. The large vessels and pulmonary arteries were excluded from the ROIs. This process was repeated for each contiguous transverse level until the entire tumor had been covered by two surgeons (A.I. and S.K.).

For the volumetric analysis of lung nodules, CT values ranging from −800 to −300 were defined as GGO regions, while values greater than −300 were defined as solid parts, according to the default settings in the SYNAPSE VINCENT software program. This 3D volumetric analysis provided measurements of the total tumor volume, solid component volume, and nonsolid component volume. The C/T ratio was calculated by dividing the consolidation diameter by the entire tumor diameter, whereas the 3D‐C/T ratio was determined by dividing the consolidation volume by the entire tumor volume.

### 2.4 Pathological Evaluations

All patients underwent pulmonary resection, which included wedge resection, segmentectomy, or lobectomy. Two pathologists (K.M. and K.U.) examined the slides of each patient. In case of discordance, a consensus was reached through discussion. The histological diagnosis was made according to the guidelines outlined in the 2015 WHO classification [11]. The evaluation of tumor invasion in the pleura, vessels, airways, or lung parenchyma was performed using hematoxylin and eosin staining with elastic stain (Elastic Van Gieson). To minimize the occurrence of iatrogenic alveolar collapse as much as possible, all specimens were freshly received, injected, and fixed in 10% neutral buffered formalin in high volume. Microscopically, alveolar collapse was observed in adenocarcinomas with a lepidic growth pattern, which revealed a reduction in air with an increase in elastin and collagen fibers. Furthermore, the tumor cells did not invade the vessels, septa, or bronchi.

### 2.5 Statistical Analyses

The baseline characteristics of the different groups were summarized using the median value, along with the minimum and maximum ranges. Categorial variables were compared using Fisher's test or chi‐square test, as appropriate. Continuous variables were analyzed using student's *t*‐test. Univariate and multivariate logistic regression analyses were performed to assess the association with the presence of invasive lesions. The odds ratio (OR) with the 95% confidence interval (CI) was calculated. Receiver operating characteristic (ROC) analyses were conducted by R package “pROC”. The cutoff values were defined as those for which the Youden index was maximal. The DeLong's test was used for comparing the ROC curves. Data were analyzed using SPSS software program (IBM Japan, Tokyo, Japan), and ROC analyses were performed using RStudio (version 4.4.3; RStudio Team, Boston, MA, USA). Statistical significance was defined as *p* < 0.05.

## 3 Results

Among 517 patients diagnosed with lung adenocarcinoma, the 161 who had been preoperatively diagnosed with clinical stage IA1 were included in this study (4 AAH, 46 AISs, 27 MIAs, and 84 IAs). Subsequently, we categorized the patients into two groups: the non‐invasive lesion group (AAH‐AIS) and the invasive lesion group (MIA‐IA).

Comparisons of the clinical and radiological characteristics of patients in the non‐invasive and invasive lesion groups are summarized in Table 1. The cohort comprised of 84 males and 77 females with ages ranging from 25 to 90 (mean age, 72) years old. Among the 161 patients, 74 had a history of smoking. The mean tumor diameter and consolidation diameter on CT were 16.0 (5.0–52.0) mm and 5.0 (0.0–14.0) mm, respectively. Surgical procedures were lobectomy in 20 cases, segmentectomy in 67 cases, and wedge resection in 72 cases. Compared to the non‐invasive lesion group, the invasive lesion group was male‐dominant and had a larger consolidation diameter, C/T ratio, consolidation volume, and 3D C/T ratio, as well as higher SUVmax. However, no significant differences were observed in age, smoking history, tumor diameter, tumor volume, or CEA.

**TABLE 1:** Patient characteristics.

	Total	Non‐invasive lesion (AAH‐AIS)	Invasive lesion (MIA‐IA)	*p*
	(*N* = 161)	(*N* = 50)	(*N* = 111)	
Age (years, range)	72 (25–90)	72 (25–87)	72 (41–90)	0.128
Sex				**0.038**
Male	84	20	64	
Female	77	30	47	
History of smoking				0.206
Yes	74	16	58	
No	47	15	32	
Missing data	40	19	21	
Tumor diameter (mm, range)	16.0 (5.0–52.0)	16.0 (6.0–39.0)	16.0 (5.0–52.0)	0.31
Consolidation diameter (mm, range)	5.0 (0.0–14.0)	3.5 (1.0–14.0)	6.0 (0.0–10.0)	**< 0.001**
C/T ratio	0.31 (0.0–1.0)	0.24 (0.05–1.0)	0.35 (0.0–1.0)	**< 0.001**
Tumor volume (mm^3^, range)	1243.0 (124.6–28012.5)	990.4 (133.3–12455.0)	1489.3 (124.6–29012.5)	0.242
Consolidation volume (mm^3^, range)	179.5 (2.9–5276.9)	94.3 (2.9–763.7)	258.9 (7.8–5276.9)	**< 0.001**
3D C/T ratio	0.166 (0.002–1.000)	0.069 (0.002–0.517)	0.203 (0.005–1.000)	**< 0.001**
SUV_max_ (range)	1.4 (0.0–18.8)	1.16 (0.0–5.5)	1.55 (0.0–18.8)	**0.006**
CEA (ng/mL, range)	2.2 (0.4–20.0)	2.0 (0.6–14.5)	2.3 (0.4–20.0)	0.266
Procedure				**0.020**
Wedge resection	72	30	42	
Segmentectomy	67	16	51	
Lobectomy	20	3	17	
Misssing data	2	1	1	
Histologic classification				**< 0.001**
AAH	4	4	0	
AIS	46	46	0	
MIA	27	0	27	
IA with predominant lepidic component	36	0	36	
IA without predominant lepidic component	43	0	43	
Invasive mucinous adenocarcinoma	5	0	5	

*Note:* Bold value indicates statistically significant differences.

Abbreviations: AAH, atypical adenomatous hyperplasia; AIS, adenocarcinoma in situ; MIA, minimally invasive adenocarcinoma; IA, invasive adenocarcinoma; C/T ratio, consolidation/tumor ratio; 3D C/T ratio, three‐dimensional consolidation/tumor ratio; *N*, number.

The results of the univariate logistic regression analysis for assessing association with the presence of invasive lesions are presented in Table 2. The consolidation diameter (OR:1.296 [1.124–1.493], *p* < 0.001), C/T ratio (OR: 10.808 [2.134–54.742], *p* = 0.004), consolidation volume (OR:1.004 [1.002–1.006], *p* < 0.001), and 3D C/T ratio (OR:195.418 [13.476–2833.7], *p* < 0.001) were significant predictors of the presence of invasive lesions in the univariate analysis. A multivariate analysis showed that the consolidation volume was the only predictor of invasive lesions (OR:1.002 [1.000–1.005], *p* = 0.045).

**TABLE 2:** Univariate and multivariate logistic analysis.

Variables	Univariate OR (95% CI)	*p*	Multivariate OR (95% CI)	*p*
Consolidation diameter	1.296 (1.124–1.493)	**< 0.001**	1.056 (0.836–1.334)	0.648
C/T ratio	10.808 (2.134–54.742)	**0.004**	2.337 (0.100–54.498)	0.597
Consolidation volume	1.004 (1.002–1.006)	**< 0.001**	1.002 (1.000–1.005)	**0.045**
3D C/T ratio	195.418 (13.476–2833.7)	**< 0.001**	13.585 (0.463–398.6)	0.130

*Note:* Bold value indicates statistically significant differences.

Abbreviations: C/T ratio, consolidation/tumor ratio; 3D C/T ratio, three dimensional consolidation/tumor ratio; OR, odds ratio; CI, confidence interval.

**FIGURE 2:**
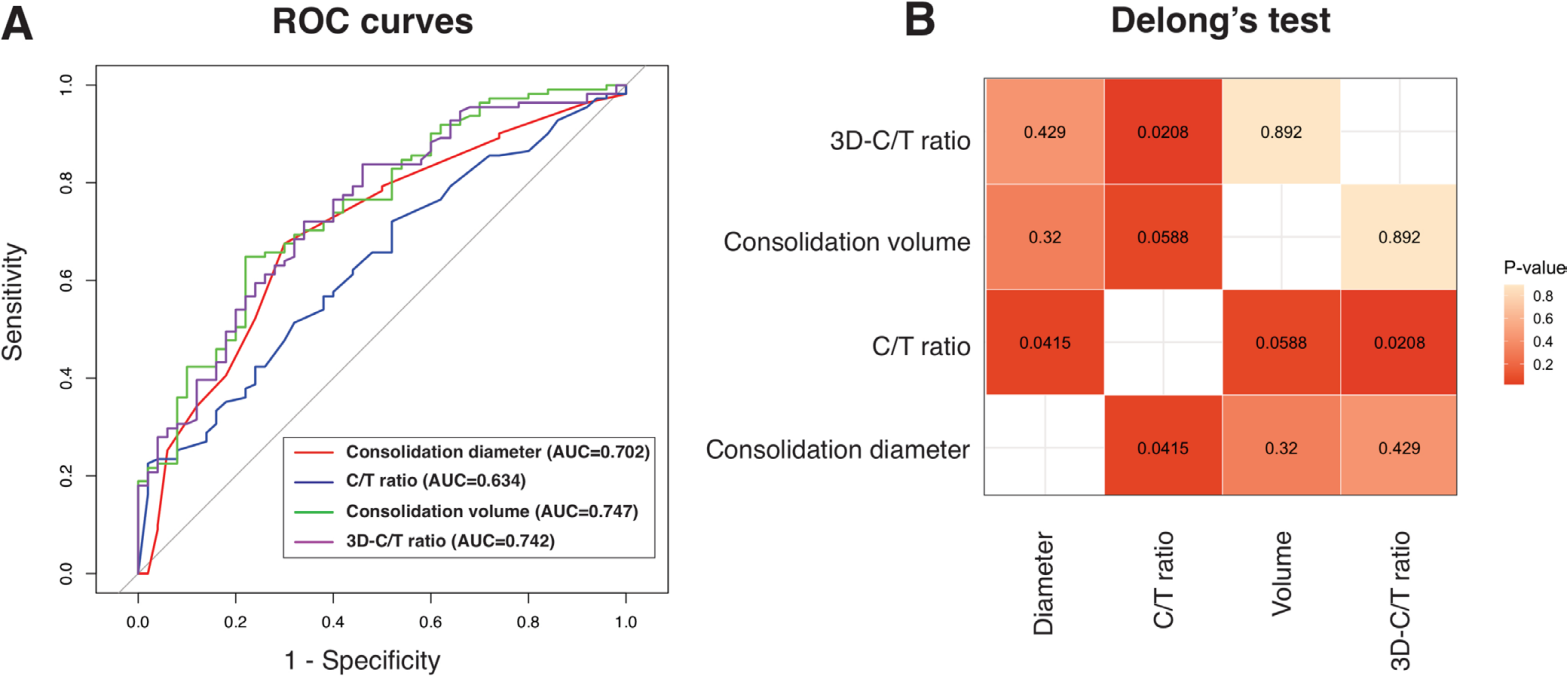
Receiver operating characteristics (ROC) curves of parameters for predicting pathological invasive lesions. (A) ROC curve for consolidation diameter, consolidation volume, consolidation/tumor (C/T) ratio, and three‐dimensional‐C/T ratio showing the area under the curve for differentiation between the preinvasive lesion group and the invasive lesion group. (B) Heatmap of the *p*‐value from comparing ROC curves for consolidation diameter, consolidation volume, C/T ratio, and 3D‐C/T ratio by Delong's test.

ROC curves were constructed for a diagnostic analysis to distinguish between the non‐invasive and invasive lesion groups. The areas under the curve (AUCs) of the consolidation diameter, C/T ratio, consolidation volume, and 3D‐C/T ratio were determined to be 0.702, 0.634, 0.747, and 0.742, respectively (Figure 2A). The AUCs for consolidation diameter, consolidation volume, and 3D‐C/T ratio were all higher than that of the C/T ratio. Furthermore, DeLong's test demonstrated significant differences between consolidation diameter and C/T ratio (*p* = 0.0415), as well as between 3D‐C/T ratio and C/T ratio (*p* = 0.0208) (Figure 2B). These findings suggest that consolidation diameter, consolidation volume, and 3D‐C/T ratio are more reliable imaging measures for identifying invasive pathological lesions than the C/T ratio in early‐stage lung adenocarcinoma. The optimal cutoff values were determined to be 4.5 mm for the consolidation diameter (specificity 70.0%, sensitivity 67.6%), 173.4 mm^3^ for the consolidation volume (specificity 78.0%, sensitivity 64.9%), and 0.121 for the 3D‐C/T ratio (specificity 66.0%, sensitivity 72.1%).

**FIGURE 3:**
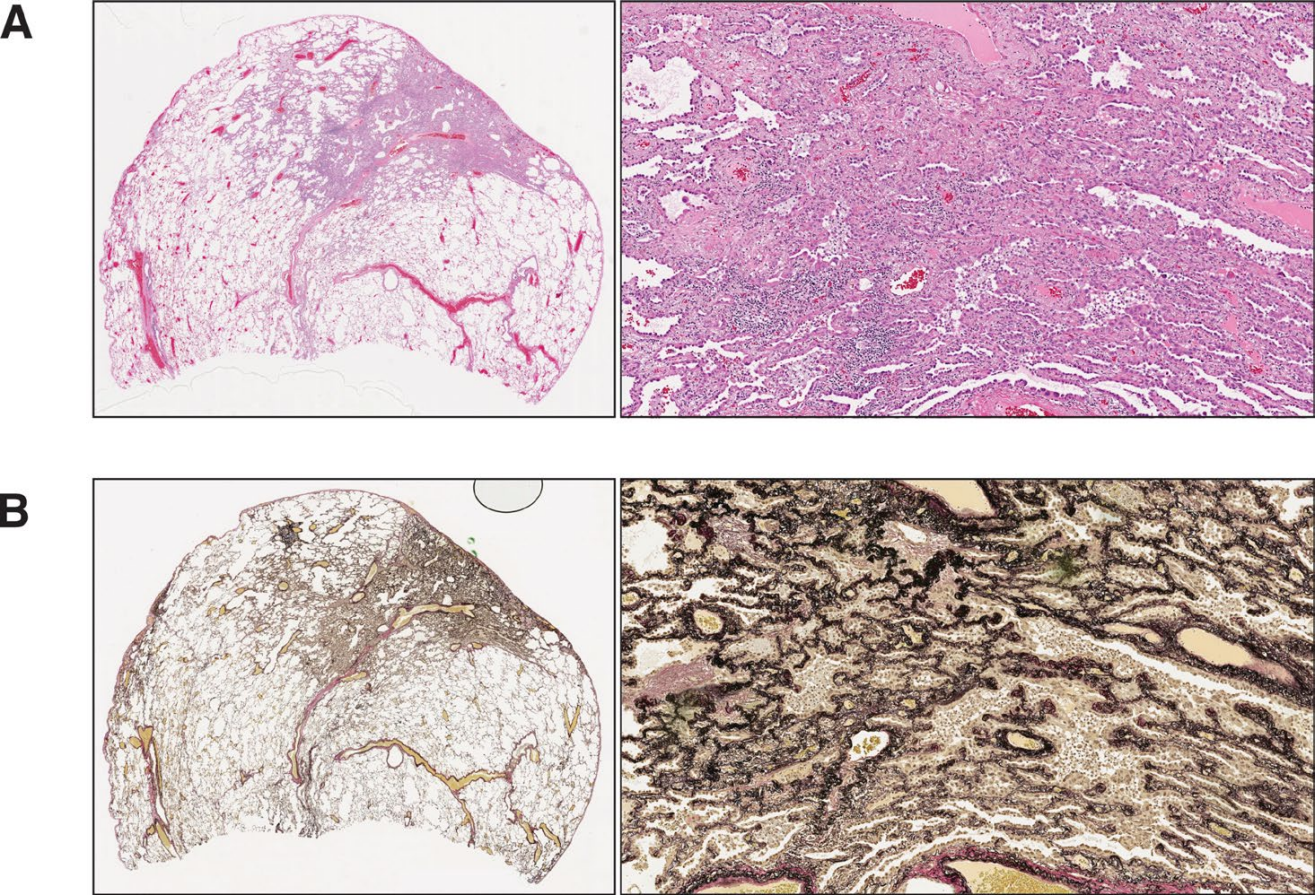
Typical pathology of alveolar collapse. (A) Histopathological specimen showing adenocarcinoma in situ with alveolar collapse (Hematoxylin–Eosin, original magnification). (B) The histopathological component of alveolar collapse indicates elastic fiber and collagen fiber growth (Elastica van Gieson, original magnification ×100).

**FIGURE 4:**
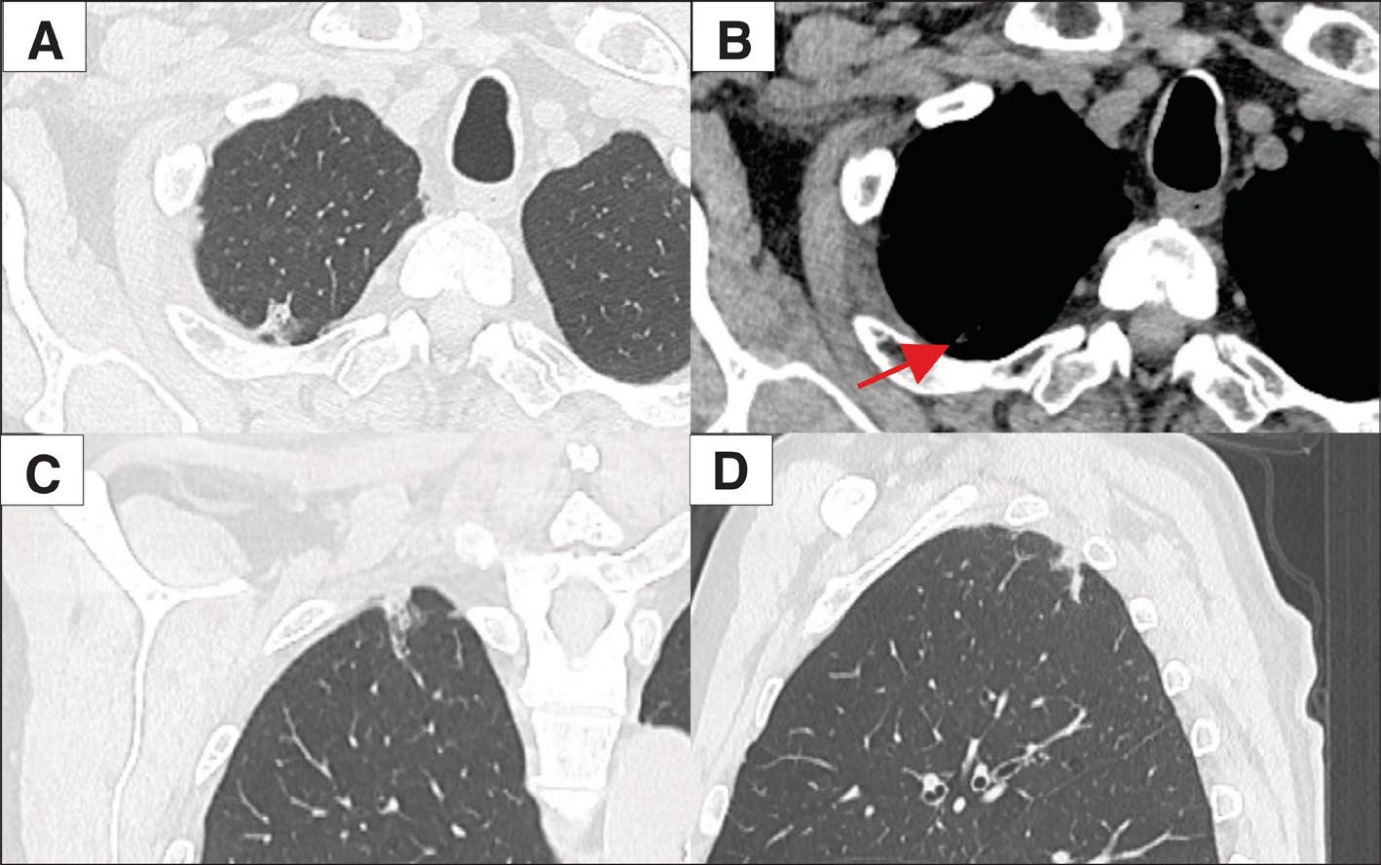
CT findings of a case of alveolar collapse in the preinvasive lesion, which contributed to the preoperative misdiagnosis of clinical stage IA1 lung cancer. Alveolar collapse is observed as a consolidation area in the mediastinal window (arrow). (A) View of the axial lung window, (B) axial mediastinal window. (C) Coronal view, (D) sagittal view.

To further characterize the pathological features of the consolidation areas in the 28 patients with non‐invasive lesions, whose samples were collected between 2019 and 2021, we performed a histological review (Figure 3). Alveolar collapse was identified in 89.2% of cases (25 of 28) (Table 3). These collapses were observed as consolidation areas on CT (Figure 4), contributing to the preoperative misdiagnosis of clinical stage IA1 lung cancer. Furthermore, alveolar collapse was found to contain elastic fibers in 96% of cases (24 out of 25) and collagen fibers in 76% of cases (19 out of 25). Alveolar collapse, composed of both elastic and collagen fibers, was present in 76% of cases (19 out of 25). Among these cases, four exhibited an increase in these fibers around the blood vessels within the tumor.

**TABLE 3:** Histological evaluation of alveolar collapse.

Non‐invasive tumor (AAH‐AIS) *N* = 28	
Alveolar collapse	
Absent	3/28 (10.8%)
Present	25/28 (89.2%)
Alveolar collapse component	
Elastic fiber	24/25 (96.0%)
Collagen fiber	19/25 (76.0%)
Both of elastic fibers and collagen fibers	19/25 (76.0%)

Abbreviations: AAH, atypical adenomatous hyperplasia; AIS, adenocarcinoma in situ.

Taken together, our findings suggest that consolidation diameter, consolidation volume, and 3D‐C/T ratio are more reliable predictors of pathological invasive lesions in early‐stage lung adenocarcinoma than C/T ratio. Furthermore, the consolidation areas in non‐invasive lesions are the result of alveolar collapse, composed of elastic and collagen fibers within the tumors.

## 4 Discussion

Lung adenocarcinomas exhibit a wide range of histological subtypes, from precursor or preinvasive to invasive lesions, each exhibiting distinct oncological behaviors that require comprehensive therapeutic management. Well‐established pathological invasive factors, including lymphatic permeation, blood vessel invasion, pleural invasion, and lymph node metastasis, have been identified as contributors to an increased rate of recurrence and an unfavorable prognosis [12, 13, 14]. Previous studies have reported that the size of solid components, rather than the size of the entire tumor, reflects the pathobiological features of the tumor [15, 16, 17]. Therefore, we aimed to assess which imaging measurement can accurately predict the presence of invasive lesions in early‐stage lung adenocarcinoma.

To date, the correlation between radiology and pathology has been investigated by the Japan Clinical Oncology Group (JCOG). The JCOG 0201 trial demonstrated that a C/T ratio of ≤ 0.25 with a tumor diameter of 2.0 cm predicted pathologic noninvasiveness (specificity 98.7%, sensitivity 16.2%) [15, 16]. Furthermore, the subsequent JCOG 0804 trial showed that patients who met these criteria, denoted as noninvasive lung cancer, exhibited no evidence of recurrence or death 5 years or longer after undergoing limited resection [18]. These studies have clarified the radiological criteria for noninvasive lung cancer and have facilitated the surgical indication for limited resection. However, it is important to note that the JCOG 0201 trial displayed low sensitivity in predicting non‐invasiveness. Among the 254 lesions with a C/T ratio ≥ 0.25, which were considered invasive adenocarcinoma on CT, 176 were later determined to be pathologically non‐invasive cancers. This overestimation may be attributed to the presence of alveolar collapse in the precursor or preinvasive lesions. Similar to the previous study, 50 of 161 patients (31.1%) were diagnosed with AAH or AIS after surgery in our study, despite having received a preoperative diagnosis of clinical stage IA1 lung cancer.

Historically, the progression of lung adenocarcinoma has been thought to follow linear multistep progression. Precursor lesions, such as AAH, progress to preinvasive AIS, followed by IA [19]. However, Yatabe et al. recently proposed a new concept suggesting that the occurrence of such linear progression depends on the specific molecular expression of adenocarcinoma [19, 20]. Their research revealed that KRAS mutations decrease in frequency during the progression from the precursor lesion to IA, whereas tumors with EGFR mutations are evenly distributed at each stage of progression [19, 20]. These findings suggest that, unlike EGFR‐mutated AAH, KRAS‐mutated AAH rarely progresses to more advanced tumor stages. According to their concept, certain precursor lesions may follow an indolent course, even without treatment; that is, surgical treatment for these lesions may be slightly over‐indicated.

Our study revealed that the consolidation diameter, consolidation volume, and 3D‐C/T ratio were significant predictors of the presence of invasive lesions. Furthermore, our multivariate logistic analysis showed that the consolidation volume was an independent predictor of pathological invasiveness. In addition, when evaluating the ROC curves, we found notably higher AUCs of consolidation diameter, consolidation volume, and 3D‐C/T ratio compared to those of the C/T ratio. To elucidate the pathological characteristics of the consolidation areas on preoperative CT in precursor or non‐invasive lesions, we performed histologic revision in the 28 patients diagnosed with AAH or AIS. This pathological reassessment revealed that 89.2% of cases (25 out of 28) exhibited alveolar collapse, which was presumably observed as consolidation on preoperative CT.

Collapse is described as the loss of air in alveolar spaces due to interstitial thickening accompanied by fibrotic foci [5, 6, 21]. Although elastin and collagen are the principal components of the connective tissue network, lung collapse has been recognized as an alveolar framework within condensed elastic fibers rather than collagen fibers [5, 22]. Therefore, elastin staining helps distinguish alveolar collapse from tumor invasion, in which elastic tissue is disrupted [23]. Similar to previous reports, we found that the main component of the collapse was elastic fibers (85.7%) rather than collagen fibers (67.8%). However, our study could not capture the pathological and morphological characteristics of alveolar collapse, which may explain why consolidation diameter, consolidation volume, and 3D‐C/T ratio were more reliable predictors for distinguishing invasive lesions from alveolar collapse than the C/T ratio. According to a previous study, a well‐defined border, concentrated distribution, and homogeneity of the solid part were characteristics of alveolar collapse on CT [4]. Additionally, 3D quantitative analysis of density uniformity and edge characteristics seems attractive candidates for further investigation. However, such subtle imaging differences are typically difficult for human observers to capture reliably. Therefore, we plan to utilize machine leaning approach to detect and quantify the 3D imaging differences between alveolar collapse and invasive lesions in the future study.

This study had several limitations that should be acknowledged. First, this study was retrospective and conducted in an academic hospital. Therefore, the possibility of unintentional selection bias cannot be excluded. Furthermore, the number of patients included in this study was relatively small. Such limited numbers restrict the generalizability and robustness of the conclusions. Therefore, validation of our findings through multicenter collaboration is essential in the future study. Second, the number of observers involved in the visual assessment of the 3D volumetric analysis was limited. Additionally, we could not perform any intra‐ or inter‐observer consistency test to assure the reliability of measuring tumor diameter and volume. Finally, intraoperative deflation may have contributed to alveolar collapse. However, it is imperative to clarify that we promptly perfused the bronchial and transpleural areas with formalin after lung resection to minimize the potential impact of iatrogenic or mechanical collapse.

In conclusion, we found that consolidation diameter, consolidation volume, and 3D‐C/T ratio are more reliable predictors of pathological invasive lesions than the C/T ratio in early‐stage lung adenocarcinoma. Furthermore, alveolar collapse can cause consolidation of partially solid GGNs in early‐stage lung cancer. Importantly, accurately distinguishing alveolar collapse from invasive lesions may contribute to more appropriate therapeutic decision‐making and ultimately improve patient outcomes.

## Author Contributions

**Shinji Kaneda, Atsushi Ito:** conceptualization, methodology, investigation, formal analysis, and writing – original draft preparation. **Daisuke Ito, Teruhisa Kawaguchi, Motoshi Takao:** writing, review, and editing. **Koji Kawaguchi:** writing, review, editing, and supervision.

## Funding

This work was supported by JSPS KAKENHI Grant Number JP20K09162.

## Ethics Statement

This study was approved by the Clinical Research Ethics Review Committee of Mie University Hospital (Ethics approval number: H2021‐133).

## Consent

This study was conducted using an opt‐out approach. Information about the study, including its purpose and procedures, was made publicly available, and participants were given the opportunity to decline participation.

## Conflicts of Interest

The authors declare no conflicts of interest.

## Data Availability

The data that support the findings of this study are available from the corresponding author upon reasonable request.
